# Current dilemma and future directions over prophylactic cranial irradiation in SCLC: a systematic review in MRI and immunotherapy era

**DOI:** 10.3389/fonc.2024.1382220

**Published:** 2024-07-30

**Authors:** Lingrong Tang, Guangwei Tian, Nan Li

**Affiliations:** Department of Radiation Oncology, the First Hospital of China Medical University, Shenyang, China

**Keywords:** small cell lung cancer (SCLC), prophylactic cranial irradiation, immunotherapy, hippocampal protection, neurocognitive

## Abstract

Small cell lung cancer (SCLC) is the most malignant pathological type of lung cancer with the highest mortality, and the incidence of brain metastasis (BM) is in high frequency. So far, prophylactic cranial irradiation (PCI) has been suggested as an effective treatment for preventing brain metastasis of SCLC. PCI has long been applied to limited-stage SCLC (LS-SCLC) patients who have achieved complete remission after radiotherapy and chemotherapy as a standard treatment. However, the neurocognitive decline is a major concern surrounding PCI. New therapeutic approaches targeting PCI-induced neurotoxicity, including hippocampal protection or memantine, have been increasingly incorporated into the therapeutic interventions of PCI. Helical tomotherapy, RapidArc, and Volumetric-modulated arc therapy (VMAT) with a head-tilting baseplate are recommended for hippocampal protection. Besides, in the MRI and immunotherapy era, the significance of PCI in SCLC patients is controversial. SCLC patients with PCI should be recruited in clinical trials since this is the only way to improve the existing standard of care. This review summarizes the current therapeutic strategy and dilemma over PCI for SCLC, providing a theoretical basis for clinical decision-making and suggestions for PCI practice in clinical.

## Introduction

Small cell lung cancer is highly invasive and malignant as a neuroendocrine cancer. It tends to metastasize, and brain metastases (BM) are the most common distant metastases (1). In 80% of patients with SCLC, BM is developed within two years of diagnosis, which seriously affects the quality of life and survival. Due to the advancement of treatment technologies and standardized treatment methods, the survival times of SCLC patients are prolonged (2–4). Prophylactic cranial irradiation (PCI) has been suggested as an effective treatment to prevent the development of brain metastasis after SCLC (5, 6). However, there has been a growing awareness of the negative impact of PCI on neurocognitive function (NCF).With the advancement of radiotherapy technologies, PCI for hippocampal protection has become a hot topic due to the low incidence of hippocampal brain metastasis and the critical function of the hippocampus in neurocognition. In addition, there are more clinical strategies, such as applying drugs like memantine and donepezil and implementing specific radiotherapy methods. This review summarizes the current therapeutic strategy and dilemma over PCI for SCLC, providing a theoretical basis for clinical decision-making and suggestions for PCI practice in clinical.

## The challenged position on PCI for SCLC

In 1999, a meta-analysis summarized seven randomized trials, including 987 SCLC patients with either limited-stage SCLC (LS-SCLC) or extensive-stage SCLC (ES-SCLC) who received complete remission. The incidence of brain metastasis and mortality were significantly reduced in the PCI group (7). In 2001, another meta-analysis evaluated 1547 patients in 12 randomized trials and reported almost the same results. The results established the value of PCI as the standard treatment for SCLC patient who was in complete remission (8). For resectable small-cell lung cancer, Yang et al. conducted a meta-analysis. They found that PCI can reduce the incidence of brain metastasis and improve the overall survival(OS) rate in stage II−III, except for pathology stage I (9). In the era of magnetic resonance imaging (MRI), for LS-SCLC, the retrospective analysis from MD Anderson in 2020 revealed that PCI does not improve overall survival and brain metastasis rates (10). The NCCN guidelines in 2022 noted that the benefits of PCI in SCLC patients with pathological stage I-IIa (T1-2N0M0) are unclear. And so are the ASTRO and ESMO clinical practice guidelines. MRI surveillance may be used as an alternative.

For ES-SCLC, the role of PCI in patients who respond to systemic chemotherapy is still debatable. The results of current randomized studies are different. The European Organization for Research and Treatment of Cancer (EORTC) conducted the first phase III study in 2007 (11). The study enrolled 286 Patients with extensive disease SCLC who responded to first-line chemotherapy. The results demonstrated that the PCI Group had a decreased 1-year brain metastase rate by 25.8% (14.6% in the PCI group and 40.4% in the clinical observation group, P < 0.001), and the 1-year OS increased by 13.8% (27.1% in the PCI group and 13.3% in the clinical observation group, P = 0.003). Therefore, PCI has become the standard of care for SCLC patients who respond to the initial treatment. The results of the EORTC study were critiqued for not necessitating routine imaging examination to determine whether there was brain metastasis (12). In another trial conducted in Japan, 224 patients with at least partial remission after initial treatment were randomly divided into PCI or no PCI groups (13). In this trial, brain MRI was required before enrollment to confirm without BM at baseline. Furthermore, all patients were required to have an MRI of the brain surveillance every three months for one year and again at 18/24 months. The OS benefit was not confirmed in this study. This Japanese study also found decreased brain metastases in the PCI group. Due to the inconsistent conclusions of two heavyweight papers, NCCN changed the evidence level of PCI to 2A. In the era of MRI, the prevalence of detected brain metastasis was 24%, which was significantly higher than 10% in the era of CT. So, a Brain MRI is essential before PCI. In 2021, a study reported the results of the utility of PCI for ES-SCLC in MRI screening. They identified nearly 16% of unsuspected brain metastases near the completion of chemotherapy (14). In this retrospective cohort, PCI was associated with a significant reduction in brain metastases in one year without a demonstrable OS benefit. A meta-analysis presented in 2023 ASCO showed that administration of PCI is associated with a significant OS benefit, but not when considering studies that radiographically confirmed the absence of intracranial metastasis at restaging or randomized controlled trials, suggesting that patient selection may contribute to this observation (15). In the aspect of cost-effectiveness, PCI is not superior to MRI surveillance (16). More RCT research is needed to confirm the role of PCI in the era of MRI screening.

## The toxicity of PCI

The toxicity of PCI is mainly divided into acute and long-term toxic effects. Acute toxic effects include fatigue, hair loss, red scalp, headache, and mild nausea (mainly low-level toxicity). Fatigue and hair loss are the most common short-term toxic effects. The primary manifestation of long-term toxicity is neurocognitive decline, such as intellectual impairment, abnormalities in brain imaging, and dementia or ataxia (17–20). With the improvement of treatment technology, including immunotherapy, the survival time of SCLC patients is prolonged. The damage to neurocognitive function caused by PCI has attracted more and more attention.

Radiation-induced brain injury begins with radiation-induced vascular injury, resulting in a change in capillary permeability and vascular edema. Circulating endothelial cells are specific and sensitive indicators of vascular injury. Besides endothelial injury, radiation damages astrocytes, oligodendrocytes, and neural progenitor cells. Demyelination and necrosis are typical pathological changes in the late stage of radiation-induced brain injury. Neural progenitor cells are important for neurogenesis and gliogenesis, especially in brain regions with neurogenic potential (21). The hippocampus is the most crucial region with many progenitor neuroglial cells. After radiation injury, the microenvironment of the hippocampus is destroyed, which affects the regeneration of neurons in the hippocampus, resulting in the difficulty of reversing brain injury. Radiation-induced neurocognitive function decline mainly focuses on the damage to the hippocampus, potentially significantly affecting memory (22, 23). Recently, a study of a mouse model of whole-brain radiation(WBI) found that capillary RBC flux and capillary diameter in the white matter were significantly decreased. WBI results in persistent cerebral white matter microvascular impairment, likely contributing to WBI-induced brain injury and cognitive decline (24). The above pathophysiology mechanism could explain the neurocognitive function impairment caused by PCI.

## Risk factors of neurocognitive toxicity

The cognitive toxicity of PCI in patients with lung cancer is still a fundamental clinical problem. It is unclear which patients are at the highest risk. To inform doctors and patients, then make personalized choices for PCI, the purpose is to select only those patients who receive PCI without increased risk of cognitive toxicity. In 2020, a systematic review attempted to analyze the risk factors of neurocognitive impairment caused by PCI in lung cancer (25). This review found that age, PCI dose, and PCI frequency (twice daily instead of once daily) might be associated with cognitive impairment. In the RTOG 0212 clinical trial, age (younger or older than 60) is an additional factor in cognitive decline after PCI in LD-SCLC patients (26). In other clinical trials, the cut-off value of age as an essential factor was set as 70 or 75 years old (27). Elderly patients are at higher risk of overall toxicity and neurotoxicity after PCI and tend to have worse OS. Due to the high risk of neurocognition after PCI in the elderly, PCI in the elderly should be more cautious. High-dose (36Gy/18f) and standard-dose (25Gy/10f) were also compared in RTOG 0212. It was found that the incidence of neurocognitive impairment was higher in the high-dose group. Therefore, the standard dose is widely adopted at present. Twice-daily fractions for PCI should also be avoided as it increases the risk of neurocognitive impairment.

SCLC has strong heterogeneity and invasiveness. In diagnosis and treatment, we should pay more attention to the influence of multidisciplinary teams. Eligible hospitals should incorporate MDT in neurology to manage patients throughout the process. This trade-off between survival and quality of life makes PCI suitable for shared decision-making (SDM) (28). Patients and clinicians make treatment decisions together based on clinical evidence and patient preferences.

## Strategies to reduce PCI neurotoxicity

Higher radiotherapy doses can better control brain metastasis, but the benefit must be weighed against the risk of toxicity. The dose-response effect in PCI was analyzed in a meta-analysis. The radiotherapy doses in this meta-analysis were divided into four groups (8Gy, 24Gy-25Gy, 30Gy, and 36Gy-40Gy). The results showed a significant trend toward a lower risk of brain metastasis when the radiotherapy dose increased. They also found that earlier administration of cranial irradiation decreased the risk of brain metastasis after induction chemotherapy (*P* = 0.01) (7). Two randomized trials have studied the effect of radiotherapy dose, but neither shows the advantage of a total dose exceeding 25Gy/10fraction (26, 29). Combined with the current data, the optimal radiotherapy dose choice is still 25Gy/10fraction, and higher radiotherapy doses should be avoided.

Hippocampal protection is a strategy to reduce neural injury. A study used ^18^F-FDG PET/CT to evaluate the effects of HA-PCI on brain metabolism (30). The results showed that PCI resulted in a diffuse decrease in ^18^F-FDG brain metabolism. HA-PCI retained the metabolic activity of the hippocampus. The RTOG-0933, the first prospective phase II trial of HA-WBRT for brain metastases, demonstrated that HA-WBRT leads to significant preservation of neurocognition. In 2017, a prospective study by Kristin et al. evaluated the effect of hippocampal-protected PCI on cognitive function in LS-SCLC (31). Twenty patients with LS-SCLC were included in this study. The irradiation dose was 25 Gy/10 fractions, and the average hippocampal dose limit was < 8 Gy. After PCI with hippocampal protection, the cognitive function of the patients did not decrease significantly. This prospective study shows that preserving the hippocampus has potential benefits in limiting the neuropsychological sequelae of brain radiation, but there is a risk of failure in the spared zone. PREMER (NCT02397733) confirmed HA-PCI better preserves cognitive function in SCLC (32). The trial of SAKK 1512 showed no neurocognitive function decline 6 and 12 months after early HA-PCI, which does not appear to be better, but it is somewhat similar to the patients who receive sequential PCI without HA (33). NCT01780675, a multicenter randomized phase III trial, also did not show a lower probability of neurocognitive function decline in the HA-PCI group than in conventional PCI (34). Neither beneficial effects of HA-PCI on self-reported cognitive functioning and QoL (35). With the help of high-quality research MRI scans, hippocampal atrophy and memory were evaluated for 4 months and 12 months after (HA-)PCI in the NCT01780675 trail. HA-PCI reduces hippocampal atrophy, but hippocampal atrophy was not associated with memory decline. The usefulness of HA-PCI is still subject to debate (36). We expect the new RCT to clarify the neuroprotective effect of HA-PCI, like NCT04535739 and NRG-CC003.

**Figure 1:**
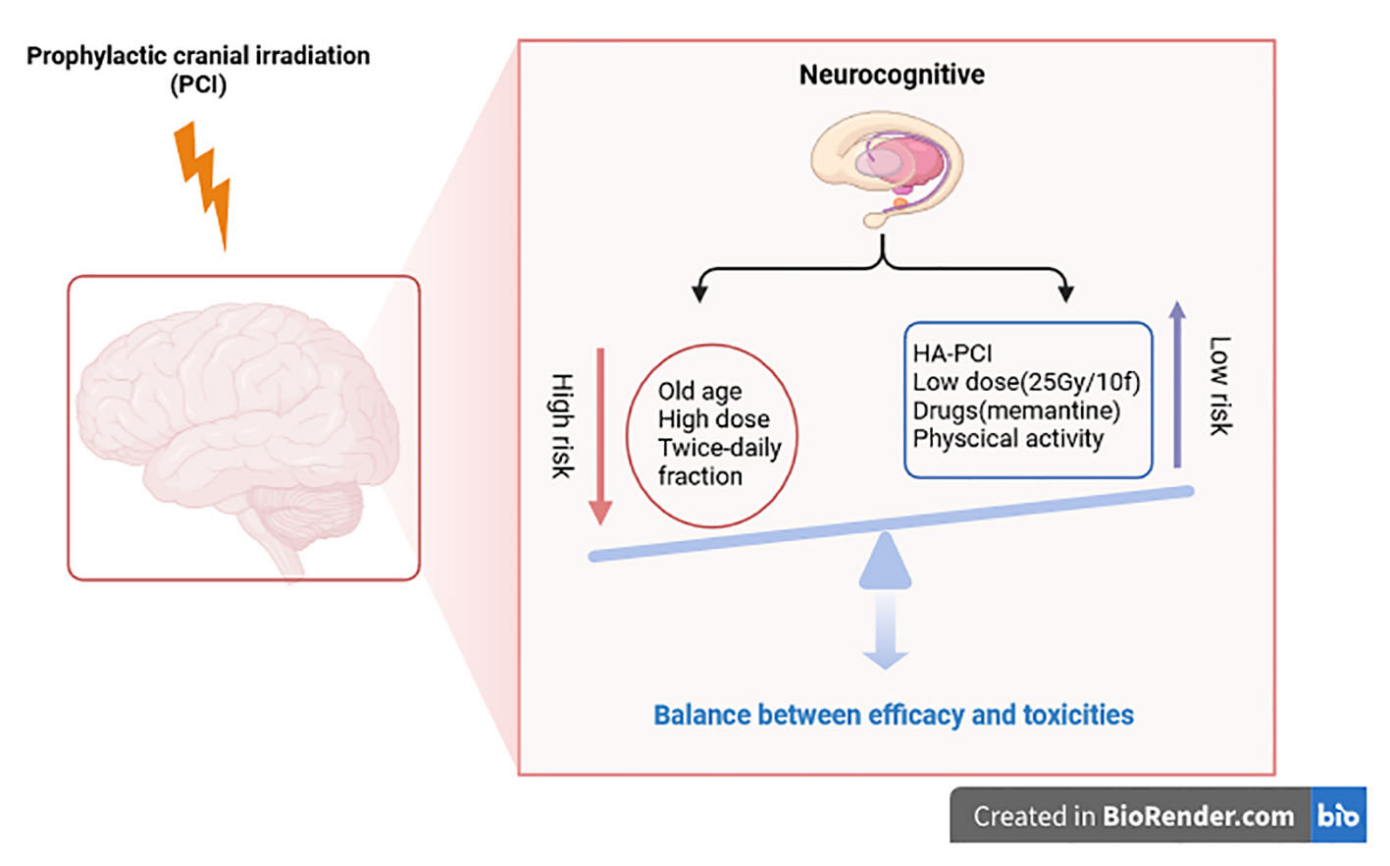
Balance between efficacy and toxicities.

The application of neuroprotective drugs is also a measure to reduce nerve injury. The prospective randomized NRG Oncology CC001 confirmed that HA-WBRT plus Memantine preserved neurocognitive function while achieving similar intracranial control and survival (37).Physical activity may reduce neurocognitive impairment after PCI. Physical activity benefits healthy adults’ cognition by inducing brain plasticity, especially in the medial temporal lobe (hippocampus). In 2021, a study from Spain was the first to use neuroimaging methods to prove that physical activity can prevent the harmful effects of systemic chemotherapy and brain radiation on the brain structure of lung cancer people, especially SCLC patients (38) (**Figure 1**).

## Radiotherapy implementation technology of HA-PCI

HA-PCI has been demonstrated safety and feasibility and has been widely used in clinical settings. In addition to the critical role of the hippocampus in neurocognition, the brain metastasis rate in the hippocampus is relatively low, making HA-PCI possible. Gondi et al. retrospectively analyzed 371 patients with 1133 brain metastases. Metastases within 5 **mm** around the hippocampus accounted for 8.6% of patients, 3% of the total brain metastases, and no metastasis in the hippocampus (39). Harth et al. identified 100 patients with 856 brain metastases, 0.4% in the hippocampus, and 3% in all patients (40). Similarly, Wan et al. included 488 patients with lung cancer and 2270 metastases. Among all brain metastases, hippocampal metastasis was seven patients (0.3%). Among the hippocampal metastases, one patient (14.3%) had oligometastatic metastasis, and six patients (85.7%) had non-oligometastatic metastasis (41). Ghia et al. analyzed 100 patients with brain metastasis, a total of 272 brain metastases. In these 100 patients, the metastasis rate within 5 **mm** around the hippocampus was 3.3%, and the metastasis rate beyond 15 **mm** around the hippocampus was 86.4% (42). It shows that the regional metastasis rate within 5 **mm** around the hippocampus is low, and most metastatic lesions are far from the hippocampus. At the same time, it is also recommended that it is more appropriate to expand 5 **mm** around the hippocampus when outlining the hippocampal region.

Hippocampus contours of HA-PCI were drawn based on the MRI scans by physicians strictly following the RTOG 0933 contouring guidelines. The dosimetric criteria of HA-PCI trials in SCLC are listed in **Table 1**. The dose and fractions were 25Gy/10fractions. The plan was designed by helical tomotherapy or IMRT, or VMAT. With the progress of radiotherapy equipment, the implementation of HA-PCI has gradually developed, such as IMRT, VMAT, and HT. Some scholars analyzed the advantages of different radiotherapy equipment and technologies for HA-WBRT. Graham showed that the MRI-guided radiotherapy (MRgRT) treatment plan using an MRI-guided linear accelerator is comparable in quality and delivery accuracy to non-MRI-guided C-Arm linac plans (43). Although WBRT and PCI differ in therapeutic purpose and dosage, they are similar in treating entire intracranial contents, as are the techniques used to protect the hippocampus. Due to its superior dose distribution, tomotherapy was considered the preferred method of HA-WBRT, but its’ treatment duration is significantly longer than co-planar VMAT (44, 45). Compared to coplanar VMAT, RapidArc can provide good dose distribution when tomotherapy cannot be performed (46). Proton beam therapy (PBT) is one of the most advanced radiotherapy methods in tumor therapy. Compared with VMAT, Dmean in the bilateral hippocampus was on average reduced from 4.6 Gy to 3.2 Gy (p < 0.001) and D40% from 3.5 to 1.2 Gy (p = 0.001) when using intensity-modulated proton therapy (IMPT). The results showed that IPMT has significant advantages in hippocampal protection. IMPT may be included in future prospective studies (47).

**Table 1:** Dosimetric Criteria of HA-PCI Trials in SCLC.

Trial	Phase	Dose/Fraction	Hippocampus	PTV	Plan
PREMER (NCT02397733)	III	25Gy/10f	Optimum D_100%_ <9 Gy D_max_<16 Gy Acceptable D_100%_ <10 Gy D_max_ <17 Gy	Optimum D_2%_: 26.7 Gy D_98%_: 23.7 Gy Acceptable D_2%_: 31.2 Gy D_98%_: 20.7 Gy	IMRT or VMAT
NCT01780675	III	25Gy/10f	D_mean ≤_8.5Gy D_1%_ ≤10Gy	D_max_<28.75(115%) V_115%_<1%	IMRT
SAKK 1512	II	25Gy/10f	D_98%_ ≤10 Gy D_max ≤_10 Gy	D_95_≥95%	helical tomotherapy or IMRT or VMAT
NCT01797159	II	25Gy/10f	D_mean_<8 Gy	D_90_≥90%	IMRT

However, due to their high cost, most hospitals do not have tomotherapy, RapidArc, and IMPT techniques. Improved VMAT or IMRT using a head-tilting baseplate has been studied. Siglin et al. first studied the plan using the head phantom with different tilt angles (48). They found that when the angle was tilted 30°, the maximum hippocampal dose decreased by 34%. However, this result is inconsistent with the results using real patient data. Moon et al. compared the plan using a 30° tilted head position and a non-tilted head position (49). They concluded that the maximum hippocampal dose of tilted patients’ head was reduced by 16.2% on average. Fu et al. improved the conventional planning method using VMAT and 45° of the patient’s head tilt angle to achieve a high HA-PCI treatment plan (50). The max and mean doses to the hippocampus of this improved VMAT were lower than 9 Gy and 7 Gy, respectively, similar to the tomotherapy plan.

Compared to dual-arc conventional VMAT, a radiation therapy technique with split-arc and reduced field size, named split-arc partial-field volumetric modulated arc therapy (sapf-VMAT), could significantly reduce the D100%, Dmax, mean dose in the hippocampus, without affecting PTV coverage (51).

## Advances of immunotherapy in SCLC

SCLC is an invasive disease with high metastatic potential. About 70% of patients with SCLC are in the advanced stage when they are found. For more than 30 years, the treatment of ES-SCLC has been platinum-based chemotherapy, although the treatment resistance rate and disease recurrence rate are very high, and the survival time of patients is short (52, 53). Before the emergence of immunotherapy, in particular immune checkpoint inhibitors (ICIs), people tried various treatments, but they did not significantly improve survival. However, with the exciting results of four phase III clinical trials (Impower133, CASPIAN, CAPSTONE-1, ASTRUM-005) and improved prognosis of patients, immunotherapy combined with chemotherapy has been included in the first-line treatment of advanced small cell lung cancer (54–57). The results showed that the addition of programmed death-ligand 1(PD-L1) or programmed death-1(PD-1) inhibitors could improve the survival of patients. Further, the application of these immune checkpoint inhibitors was approved in the first-line treatment of ES-SCLC. However, the absolute benefit of PD-L1 or PD-1 inhibitors in SCLC is more limited than in non-small cell lung cancer. Understanding SCLC’s phenotypic and immune characteristics is essential to screening more suitable benefit populations. A recent analysis of SCLC gene expression profiles has clarified four molecular SCLC subtypes driven by global transcription regulators (ASCL1, NEUROD1, POU2F3, and YAP1), which may be the basis for determining SCLC predictive biomarkers (53, 58, 59). A subtype of inflamed has been described (SCLC-I), characterized by low ASCL1, NEUROD1, and POU2F3 expression, and is predicted to be more sensitive to ICI treatment. This study included a retrospective analysis of 133 patients, of which 18% were classified as SCLC-I subtype. In this analysis, the SCLC-I subtype benefited more from adding atezolizumab than other SCLC subtypes (58). A recent comprehensive RNA sequencing of 286 pretreatment SCLC tumor samples in CheckMate 032 was reported. This study showed that LSD1 gene expression correlates with worse survival outcomes for patients treated with either nivolumab or the combination of nivolumab and ipilimumab (60). For recurrent SCLC, single immunotherapy is approved in the third-line treatment and was mainly based on the results of these trials (KEYNOTE-028, KEYNOTE-158, and CheckMate 032) (61, 62). Immunotherapy in LS-SCLC needs better results to determine its status. The current research results cannot draw a unified conclusion in LS-SCLC.

## Current new trial and future directions of PCI in SCLC

To solve the problems about PCI still existing in SCLC, several clinical phase 3 trials are still being carried out. We searched and screened the clinical trials at the ClinicalTrials.gov database. The phase III clinical trials of PCI in SCLC are shown in **Table 2**.

**Table 2:** RCT summary of using PCI in SCLC.

Trial Number	Title	Status	Conditions	Population	Interventions	Outcome Measures	Location
NCT04535739	PCI for Patients With ES- SCLC After RCT: a Prospective Randomized Study	Recruiting	Small-cell Lung Cancer	414	Radiation: prophylactic cranial irradiation	•overall survival •progression-free survival •brain metastases rates •side effect	China
NCT04829708	Efficacy and Safety of Prophylactic Cranial Irradiation Versus MRI Surveillance in Patients With Limited-stage Small Cell Lung Cancer Who Achieved Remission After First-line Chemoradiotherapy	Recruiting	Limited Stage Small Cell Lung Cancer	534	•Other: MRI Surveillance •Radiation: Prophylactic Cranial Irradiation	•Overall survival (OS) •1-year overall survival rate (1y-OS%) •3-year overall survival rate (3y-OS%) •Progression-free survival (PFS) •Brain metastasis rate •Cumulative incidence of neurocognitive impairment	China
NCT04790253	PRophylactic Cerebral Irradiation or Active MAgnetic Resonance Imaging Surveillance in Small-cell Lung Cancer Patients (PRIMALung Study)	Recruiting	Limited Stage/Extensive-stage Small Cell Lung Cancer	600	Radiation: Prophylactic cranial irradiation	•Overall survival •cognitive failure free survival •Quality of Life •Safety profiling	Europe
NCT04155034	SWOG S1827 (MAVERICK) Testing Whether the Use of Brain Scans Alone Instead of Brain Scans Plus Preventive Brain Radiation Affects Lifespan in Patients With Small Cell Lung Cancer	Recruiting	Limited Stage/Extensive-stage Small Cell Lung Cancer	668	•Procedure: Magnetic Resonance Imaging •Radiation: Prophylactic Cranial Irradiation	•Overall survival (OS) •Cognitive failure-free survival (CFFS) •CFFS rate •Cumulative incidence of cognitive failure •OS in an “as-treated” analysis •Brain metastases-free survival (BMFS) •Incidence of adverse events	USA
NCT02635009	Whole-Brain Radiation Therapy With or Without Hippocampal Avoidance in Treating Patients With Limited Stage or Extensive Stage Small Cell Lung Cancer	Active, not recruiting	Limited Stage/Extensive-stage Small Cell Lung Cancer	392	•Radiation: 3- Dimensional Conformal Radiation Therapy •Other: Cognitive Assessment •Radiation: Intensity- Modulated Radiation Therapy •Other: Laboratory Biomarker Analysis •Other: Quality-of-Life Assessment	•HVLT-R delayed recall deterioration status, defined using the Reliable Change Index (RCI) •Intracranial relapse rate •Cost-effectiveness as measured by the EQ-5D •Incidence of adverse events (AEs), as measured by the CTCAE v.4 •Intracranial relapse rate •Overall survival •Patient-reported HRQOL, as measured by the EORTC QLQ-C30 and BN20 (Phase III) •Preservation of neurocognitive function, as measured by neurocognitive decline for HVLT-R, COWA test, TMT Parts A and B, and Clinical Trial Battery Composite (CTB COMP) score (Phase III) •Time to neurocognitive failure, where a failure is defined using the RCI criteria, as measured by HVLT-R, COWA test, and TMT Parts A and B	USA
NCT05651802	PCI and Brain MRI Follow-up vs Brain MRI Follow-up Alone in Limited-stage SCLC Patients	Recruiting	Limited-stage Small Cell Lung Cancer	220	•Radiation: Prophylactic cranial irradiation •Radiation: Thoracic radiotherapy •Drug: Chemotherapy	•2-year brain metastasis-free survival estimated with kaplan-Meier method [Time Frame: Up to 2 years] •2-year overall survival estimated with kaplan-Meier method [Time Frame: Up to 2 years] •Intra-cranial progressive patterns [Time Frame: Up to 2 years]	China
NCT02605811	Temozolomide in Preventing Brain Metastases in Small Cell Lung Cancer	Recruiting	•Small Cell Lung Cancer •Metastatic Carcinoma	426	•Drug: temozolomide •Radiation: prophylaxis cranial radiotherapy	•2-year incidence of brain metastases [Time Frame: 2 year]	China

SWOG 1827 MAVERICK (NCT04155034), a randomized phase III trial examining PCI with MRI surveillance versus MRI surveillance alone in patients with SCLC, may redefine the role of PCI for patients with SCLC in the modern era. The estimated enrollment is 668 participants. Immunotherapy concurrent with and/or adjuvant to first-line therapy is allowed at the treating physician’s discretion. The primary objective is to evaluate whether OS with MRI surveillance alone is not inferior to MRI surveillance combined with PCI for the treatment of SCLC. Secondary objectives include the following six aspects, which were shown in clinicaltrials.gov. The results of this trial may help elucidate whether PCI is essential in the immune era.

PRIMALung Study (NCT04790253), Like SWOG 1827 MAVERICK, immunotherapy concurrent with and/or adjuvant to standard therapy is also allowed in this trial. The conclusion of this RCT trial will further clarify the role of PCI in SCLC and screen the dominant population suitable for monitoring MRI to avoid PCI in the immune era.

NRG-CC003 (NCT02635009), the randomized phase II/III trial, studies how well PCI works and compares it with or without hippocampal avoidance in treating patients with SCLC. If the rate of Intracranial relapse in the HA-PCI arm is significantly greater than that of the PCI-only arm, this study will not continue to the phase III portion. The results of NRG-CC003 will answer the question regarding the benefit of HA-PCI.

NCT04535739 will answer whether patients with ES-SCLC after chemotherapy and thoracic radiation can benefit from PCI. NCT04829708 wants to compare PCI versus MRI surveillance in patients with LS-SCLC who achieved remission after first-line chemoradiotherapy. Unlike the SWOG 1827 MAVERICK trial, immunotherapy isn’t allowed and only includes LS-SCLC in this trial.

Can MRI surveillance replace PCI in the MRI and immunotherapy era? Is HA-PCI necessary? Further discussion and research are needed.

In the future, neuroimaging can provide unique, objective, and important biomarkers of cognitive changes. However, at present, no RCT includes a neuroimaging examination. Biomarkers in cerebrospinal fluid have also been studied, but they are unsuitable for routine use. Blood samples are relatively easier to obtain than cerebrospinal fluid, but no studies on patients with SCLC exist. In the SWOG 1827 MAVERICK (NCT04155034) trial, the additional objective includes collecting blood for banking. These blood will be used if appropriate specific blood markers can be detected.

In the era of Artificial intelligence(AI) intelligence, fully automated planning and delivery of HA-WBI(PCI) may be the future. It can generate plans significantly faster than VMAT plans (63).

## Some notable problems

The first problem is the risk of intracranial recurrence. Harth et al. reported that the hippocampal metastasis rate of small-cell lung cancer was relatively high (18.2% of all patients with small-cell lung cancer). The rate of hippocampal brain metastasis in non-small cell lung cancer was low (2.8%) (40). In LS-SCLC treatment, a study from Korea evaluated intracranial failure after hippocampal avoidance prophylactic head irradiation (HA-PCI). Of the 48 patients who underwent HA-PCI, two patients developed perihippocampal recurrence (64). Although HA-PCI may increase the risk of intracranial failure, HA-PCI did not affect disease control or survival (31, 32, 34, 64). Whether HA-PCI can reduce PCI-induced neurotoxicity and improve quality of life compared with conventional PCI remains controversial. Therefore, the risk of intracranial recurrence should be explained before patients choose HA-PCI. In addition, regular MRI monitoring helps detect new lesions on time and perform salvage radiosurgery.

The second one is that methods for assessing cognitive function are not standardized. Neurocognitive impairment is affected by many factors. Many patients with SCLC have neurocognitive impairment before PCI. These factors may be associated with age, the influence of chemotherapy drugs, nutritional status, anemia, etc. (20, 65) How neurocognitive detection can be more objective and sensitive in the future trial design is important for HA-PCI. In the published clinical trials, the methods of neurocognitive function detection were mainly analyzed by scale. For example, delayed free recall (DFR) on the Free and Cued Selective Reminding Test (FCSRT) was used in the PREMER trial, while the Hopkins Verbal Learning Test (HVLT)-Recall was used in RTOG 0212 and 0214 trials. In addition, some trials used QLQ-C30 and BN20 to evaluate the cognitive toxicity of PCI, which only included the field of cognitive function rather than any cognitive screening test or neuropsychological test (11, 66, 67).

TMT, HVLT, and COWAT were recommended in The International Cognition and Cancer Task Force (ICCTF) published consensus (68). In some cases, it was found that the toxicity scores from patients and doctors were not always consistent. Some studies even show that QOL data evaluated by clinicians were unreliable. Some studies’ evaluations were based on patient-reported data. However, according to the recommendations of ICCTF, objective testing is still the gold standard to measure cognitive function. Different cognitive evaluation methods may lead to the bias of the research results. Utilizing multiple assessment scales to comprehensively evaluate different cognitive domains (such as memory, executive function, attention, etc.) can provide a complete understanding of cognitive function for future clinical trials. Additionally, digital and automated cognitive assessment tools like CANTAB can offer more objective and consistent assessment results.

The third one is that the best hippocampal dose limitation is unclear. GONDI showed that bilateral hippocampal exposure >7.3Gy is associated with cognitive function (69). TSAI et al. showed that bilateral 100% hippocampal radiation dose >5.83Gy (D100%>5.83Gy) and bilateral 80% hippocampal radiation dose >6.80Gy (D80%>6.80Gy) affected patients’ cognitive function during HA-WBRT treatment (70). MA et al. ‘s study suggested that the probability of cognitive function decline caused by bilateral 50% hippocampal radiation dose >22.1Gy (D50%>22.1Gy) was 20% (71). Different studies have used different hippocampal dose limits(**Table 1**). There is no unified standard consensus on the dose limit of the hippocampus in the current RCT. Setting subgroups with different hippocampal dose limits in clinical trials and conducting subgroup analyses would be helpful in determining more precise hippocampal dose limits.

Furthermore, radiotherapy quality control of HA-PCI is also a very important problem. Image-guided radiotherapy is very suitable because the protection of the hippocampus needs to be more accurate. Accurate treatment can ensure the perfect implementation of a radiotherapy plan, making the clinical data more authentic and reliable.

## Conclusion

This review emphasizes the important but controversial role of PCI in SCLC, providing feasible suggestions for PCI practice in clinical The clinical application of PCI is facing significant challenges because of concerns about the neurotoxicity of PCI. With various neuroprotective strategies, this technology is essential to prevent brain metastasis. However, compared with non-small cell lung cancer, the survival time of SCLC patients is short. Measuring the survival rate and quality of life requires the participation of professional doctors and patients’ opinions. MRI monitoring has also become a new choice with the popularity of MRI. RCT is needed to identify those populations that need to be monitored for MRI. Clinical trials are also needed to screen out PCI beneficiaries, and individualized treatment is required for the immune era.

## Author contributions

LT: Investigation, Writing – original draft, Writing – review & editing. GT: Conceptualization, Writing – original draft. NL: Conceptualization, Supervision, Writing – review & editing.
